# Evolution of attitudes, trends and perceptions of smoking among middle and secondary school students in the Gharb Region, Morocco, 2010–2015

**DOI:** 10.4102/phcfm.v11i1.1914

**Published:** 2019-06-19

**Authors:** Samir Mounach, Fatima-Ezzahra Zahrou, Elkhansa Mahdaoui, Latifa Belakhel, Youssef C. Khazraji, Hicham El Berri

**Affiliations:** 1 National Surveillance Unit, Directorate of Epidemiology and Disease Control, Ministry of Health, Rabat, Morocco; 2 Non-Communicable Diseases Division, Directorate of Epidemiology and Disease Control, Ministry of Health, Rabat, Morocco; 3 Lalla Salma Foundation, Cancer Prevention and Treatment, Rabat, Morocco

**Keywords:** tobacco control programme, Morocco, smoking behaviour, student, perception

## Abstract

**Background:**

Tobacco use is an important public health issue. Morocco implemented a tobacco control programme, which has been ongoing among students at middle and secondary schools since 2010.

**Aim:**

This study aims to compare the trend in smoking among the programme beneficiaries with the results of the initial study conducted prior to the implementation of the programme.

**Setting:**

This study was conducted in middle and secondary schools of the Gharb Region in Morocco between 2010 and 2015.

**Methods:**

Two cross-sectional studies were conducted in 2010 and 2015 in the middle and secondary schools of the Gharb Region. Multistage cluster sampling was used. The information was collected using a self-administered questionnaire.

**Results:**

In the first study in 2010, 5312 students participated, and in the second one in 2015, 4208 students participated. The level of information on smoking and its effects was higher in 2015 (94.0%) than in 2010 (92.5%). In 2010, parents, primary schools and television and radio were more involved in student information on smoking compared to 2015. The proportion of students claiming that tobacco was not a pleasure (86.3%) and that it does not calm nerves (76.5%) was significantly higher in 2015 than in 2010. The prevalence of smoking increased significantly in 2015 (2.9%) against 2010 (1.8%).

**Conclusion:**

This study reports the general positive evolution in knowledge about smoking and its effects. Despite that the prevalence of smokers increased in 2015. The results suggest the need to address family influences on adolescent smoking and to investigate participation of schools in education and training students in tobacco dependence prevention.

## Introduction

Tobacco use is an important public health issue, and it is the single biggest avoidable cause of death and disability worldwide.^1,2^ The burden of disease caused by smoking is enormous, with more than 6 million premature deaths annually.^3^ According to the World Health Organization (WHO), tobacco causes 4.9 million deaths per year, a figure expected to rise to more than 10 million deaths per year by 2030.^4^ The health risks of tobacco smoking are well documented. Smoking causes a wide range of irreversible diseases, leading to microbial infections (such as respiratory infections, periodontitis and bacterial meningitis) as well as various cancers related to the respiratory, digestive and genitourinary systems and poses a risk of acute leukaemia.^5^ Tobacco use is also associated with delayed recovery from injuries and a higher incidence of atherosclerosis, chronic obstructive pulmonary disease, Crohn’s disease, rheumatoid arthritis and premature death.^6^ Smoking is highly prevalent among young people in some socio-economic contexts; it primarily begins in early adolescence.^7^ Smoking among adolescents has been reported to be associated with many factors that commonly play a role in the initiation of smoking, including social factors; smoking among family members, peers and teachers; psychological relaxation; and economic factors.^8^

In Morocco, the results of the Global Youth Tobacco Survey (GYTS) conducted by the Moroccan Ministry of Health in schools highlighted the need for awareness of the prevalence of youth smoking. Prevalence in the 13–15-year age group was 10.8% (in 2001), 11% (2006) and 9.5% (2010).^9^ To combat and to limit the health risks of tobacco smoking among Moroccan adolescents, in the year 2007, the Lalla Salma Foundation – Cancer Prevention and Treatment, in collaboration with the Ministry of Health and the Ministry of Education, launched a tobacco control strategy at the level of three regions: Rabat-Salé-Zemmour-Zaer, Souss-Massa-Drâa and Grand Casablanca. It was gradually extended to be a national programme in 2009–2010. The overall goal of this strategy is to provide a framework for the implementation of tobacco control measures to promote the health and well-being of Moroccan students in the future and to protect them from the harmful effects of smoking and exposure to second-hand tobacco smoke.

The activities of this programme include the placement of smoking cessation signs in all schools, training health professionals in tobacco cessation consultation and training of teachers and peer educators of health clubs established in the schools in information, education and communication on the harmful effects of tobacco consumption.

Objectives of the programme include raising awareness and changing knowledge, attitudes and practices regarding the consumption of tobacco products, protecting smokers and non-smokers, and reducing the number of users.

The follow-up of the evolution of tobacco smoking provides important information to follow the dynamic of this public health issue. Our study aims to examine the trend of smoking by comparing two cross-sectional studies, to measure change in the degree of awareness and knowledge, attitudes and practices regarding the consumption of tobacco products. The first study was conducted in 2010 before the implementation of a national programme against smoking, and the second one in 2015, 5 years after the initial implementation.

## Methods

### Type of study

Two cross-sectional studies were conducted in 2010 and 2015 among students of middle and secondary schools of the Gharb Region in Morocco.

### Duration of the study

The study lasted for 5 years, from December 2010 to December 2015.

### Eligibility criteria

To be included in the study, children had to be aged between 10 and 20 years old, must have had informed consent provided by their tutors, and had to belong to the selected middle and secondary schools in the region of Gharb-Chrarda-Béni-Hssen.

### Steering committee

A steering committee was constituted to lead and coordinate all the activities concerning the two studies. This committee included principal investigators of the study, a representative of the Ministry of Education, local authorities and parents’ union representatives of the selected schools.

### Study site

The region where the studies took place was situated in the north-west of Morocco. The population of the Gharb-Chrarda Region is 2 million inhabitants (6% of the Moroccan population); it contains both rural and urban areas, and all socio-economic classes are represented (National Statistics Department 2014). Subsequently, factors influencing smoking are the same as in other parts of the country.

### Sampling

The list of middle and secondary schools (and classes) was obtained from the Ministry of Education of Morocco.

In 2010, the study included 10% of all the schools of the region.

**Step 1:** The list of all schools was analysed by OpenEpi software (with every school corresponding to an exclusive number) using the random function; the software randomly chose 10% of the secondary schools and 10% of middle schools. There were around 100 secondary schools and 95 middle schools in the region, of which nine secondary schools and nine middle schools were selected for inclusion.

**Step 2:** All students in the selected classes who gave their consent were included in the study

In 2015, the sample calculation was done using the formula of prevalence studies^10^, with a confidence interval of 95% (0.05) and an estimate of an 80% response rate. Concerning the number of desired estimates *G* we chose to present the results by age and sex (four age groups: 11–12, 13–15, 16–17, 18 and over), which gave us a total of eight estimates.

The cluster sampling was accomplished in two steps:

- The size of the sample was divided by the number of grade levels (first, second and third year of middle school and first, second and third year of secondary school). This allowed us to reach the subpopulation (age and sex) on which the estimates would be made; the total size was divided per level.
- Then a cluster sample proportional to the size of the classes (cluster) was selected: 66 classes from 34 secondary schools and 66 classes from 50 middle schools were identified in 2015. The investigation allowed participation of all students who were present on the day of the investigators’ visit.

### Constitution of the investigation teams and their training

Three teams were designated, one for each city (Kenitra, Sidi Kacem and Sidi Slimane are the three cities of the Gharb Region). Each team was led by a supervisor and two investigators. A training session was organised for the investigators in preparation for the two studies. The aim was to explain the objectives and the methodology to all the investigators, to explain the tasks of each one. The questionnaire was reviewed following the sessions.

### Pilot test

A pilot test was conducted in four classes of different levels in a non-participant school. Its main objectives were to test the questionnaire – to estimate the average time required to administer it and to test its comprehension by the students. A debriefing meeting was organised; the study organisation and questionnaire were adapted and validated. The questionnaire was reviewed by the steering committee.

### Data collection

The identified schools were informed by their administrative authorities of the schedule of the study. Once the investigators were present in the schools, a short meeting was organised with the director and the administrative staff.

An auto administered questionnaire was used in the two studies. The same instrument was used for both studies for the most part, with some items regarding the specific objectives of each of the two studies.

For both of the studies, the questionnaire was administered by a staff of health specialists and from the Ministry of Education.

At the entry of each selected class, the objectives of the study were explained for the students and their teacher. The investigators stayed in the class to collect the questionnaires and to respond to the students’ questions, if any.

Data regarding tobacco use were collected at the beginning of the study from children in all subject groups, using a self-administered questionnaire.

We collected data on demographic characteristics, the extent of information on smoking, sources of information on smoking, perceptions of smoking and prevalence.

### Case definition

Since its implementation the programme has defined smoking as follows:

- *former smoker*: someone who hasn’t smoked for the last 6 months
- *smoker*: someone who has smoked at least one cigarette per day for the last 6 months
- *non-smoker*: someone who hasn’t smoked at least one cigarette per day in the preceding 6 months
- *second-hand smoker*: someone who has been exposed to second-hand smoke within their home or in class for the past 30 days.

### Statistical analysis

Data analysis was done by the software IBM SPSS Statistics version 20. The nominal variables were presented as proportion. A chi-square test was used to test independence between nominal variables. In the case of cells with a theoretical frequency *n* < 5, we take Fisher’s *p*-value. Two-sided *p*-values < 0.05 were considered significant.

### Ethical considerations

The purpose and the protocol of the study were presented and explained to the local authorities, regional medical representatives, school headmasters, teaching staff and parents’ union representatives in schools, who in turn explained clearly the benefits of the study to the children’s parents. Subsequently, oral consent was obtained from children and their parents or tutors, respectively, before the beginning of the survey. Volunteering parents and children were included in the study.

This study received approval from the Ministry of National Education. The ethical clearance number is 18/23.

## Results

The demographic characteristics are presented in Table 1.

**TABLE 1:** Demographic characteristics of schoolchildren enrolled in the study in 2010 and 2015.

Variables	2010		2015		p*
	*n*	%	*n*	%	
Sex					
Female	2931	55.2	1995	49.3	0.0001
Male	2380	44.8	2053	50.7	
Sum	5311	-	4048	-	
Years strata					
10–12 years	213	4.0	42	1.1	< 0.001
13–15 years	2862	54.1	1526	38.5	
16–17 years	1545	29.2	1245	31.4	
≥ 18 years	672	12.7	1155	29.1	

Note: Results are presented as frequencies and proportions, *n* (%).

* , *p*-values were determined using the chi-square test (the chi-square value was corrected for cells with a theoretical frequency less than 5).

The level of information on smoking and its harmful effects are illustrated in Table 2. The proportion of students informed about tobacco and its negative effects was higher in the 2015 study (94.0%) than the 2010 study (92.5%) (*p* > 0.05) (Table 2).

**TABLE 2:** Level of information on smoking among schoolchildren involved in the study in 2010 and 2015.

Variables	2010 study		2015 study		p*
	*n*	%	*n*	%	
Not informed	399	7.5	247	6.0	> 0.05
Informed	4913	92.5	3882	94.0	> 0.05

Note: Results are presented as frequencies and proportions, *n* (%).

* , *p*-values were determined using the chi-square test (the chi-square value was corrected for cells with a theoretical frequency less than 5).

Table 3 shows a comparison of sources of information on smoking in the 2010 and 2015 studies. Analysis of the table shows that in the 2010 study, parents, primary schools and television and radio were more involved in student information on smoking and its harmful effects compared to the study of 2015 (Table 3).

**TABLE 3:** Comparison of sources of information on smoking in 2010 and 2015.

Variables	2010		2015		p*
	*n*	%	*n*	%	
Parents					
Yes	3908	73.6	2397	62.6	< 0.0001
No	1402	26.4	1433	37.4	
Primary school					
Yes	3368	63.4	1869	49.0	< 0.0001
No	1943	36.6	1947	51.0	
High school					
Yes	3329	62.7	2412	63.4	< 0.0001
No	1982	37.3	1394	36.6	
TV and radio					
Yes	4609	86.8	3190	83.0	< 0.0001
No	702	13.2	655	17.0	

Note: Results are presented as frequencies and proportions, *n* (%).

* , *p*-values were determined using the chi-square test (the chi-square value was corrected for cells with a theoretical frequency less than 5).

Table 4 shows the perception of smoking among students in 2010 and 2015. Comparison between the indicators in the 2010 and 2015 studies on the perceptions of students about tobacco and its harmful effects shows that the prevalence of students claiming that tobacco is not a pleasure and does not calm nerves was significantly much higher in 2015 than in 2010 (86.3% and 76.5% in 2015 versus 70.9% and 56.4% in 2010, respectively). The prevalence of students informed about the harmfulness of tobacco on health was high in both studies, 95.5% in 2010 and 94.4% in 2015 (Table 4).

**TABLE 4:** Comparing students’ perceptions of smoking in 2010 and 2015.

Variables	No		Yes		p*
	*n*	%	*n*	%	
Expensive					
2010	1720	32.4	3591	67.6	< 0.0001
2015	1106	60.1	2574	69.9	
A trap					
2010	856	16.1	152	83.9	< 0.0001
2015	784	20.7	3010	79.3	
A pleasure					
2010	3766	70.9	1545	29.1	< 0.0001
2015	3032	86.3	482	13.7	
Calms the nerves					
2010	2994	56.4	2317	43.6	< 0.0001
2015	2741	76.5	842	23.5	
Disturbs others					
2010	455	8.6	4856	91.4	< 0.0001
2015	498	13.1	3304	86.9	
Bad for health					
2010	239	4.5	5072	95.5	< 0.0001
2015	224	5.6	3806	94.4	

Note: Results are presented as frequencies and proportions, *n* (%).

* , *p*-values were determined using the chi-square test (the chi-square value was corrected for cells with a theoretical frequency less than 5).

Table 5 illustrates the prevalence of smoking among students in both studies. The comparison between the 2010 and 2015 studies shows that the prevalence of students who smoked increased in 2015 (2.9%) compared to 2010 (1.8%) (Table 5).

**TABLE 5:** Comparison of smoking prevalence among students in 2010 and 2015.

Variable	2010 study		2015 study		p*
	*n*	%	*n*	%	
Non-smoker	5066	95.4	3931	93.4	> 0.05
Former smoker	150	2.8	128	3.0	> 0.05
Smoker	95	1.8	121	2.9	> 0.05

Note: Results are presented as frequencies and proportions, *n* (%).

* , *p*-values were determined using the chi-square test (the chi-square value was corrected for cells with a theoretical frequency less than 5).

Table 6 shows that 36.9% of students were exposed to smoking in their home. The students were influenced most by their best friends smoking (32.9%), followed by their parents (27.5%) and finally by their brothers and sisters (15%).

**TABLE 6:** Exposure to smoking among students in 2015.

2015	Yes		Sum
	*n*	%	
Students exposed to smoking at home	1420	36.9	3849
Students exposed to smoking by their peers	1343	32.9	4076
Parents of students are smokers	1124	27.5	4080
Brothers of students are smokers	640	15.8	4059

Note: Results are presented as frequencies and proportions, *n* (%). The results are not available in the 2010 study.

## Discussion

Worldwide, the health impact of smoking is now well documented.^11^ Smoking is one of the leading individual risk factors for the development of the most common chronic non-communicable diseases (cardiovascular, respiratory and a number of malignant diseases), for effects on infants, children and young people’s development and health, as well as for disability, premature death and environmental pollution.^12^ Reduction in smoking prevalence is therefore one of the most important public health measures that should be implemented to improve the health of Morocco’s population. This study was designed to investigate the evolution of smoking among middle and secondary school students in the Gharb Region, Morocco, between 2010 and 2015 and to have more information that can help in public health research.

Our results showed that Moroccan students have favourable knowledge of the health risks associated with tobacco smoking. Indeed, the prevalence of informed students about tobacco and its harmful effects is slightly higher in the 2015 study (94.0%) compared to the 2010 study (92.5), and the prevalence of students claiming that tobacco is not a pleasure and does not calm the nerves was significantly higher in the 2015 study than in the 2010 study (86.3% and 76.5% in 2015 versus 70.9% and 56.4% in 2010, respectively). Moreover, the prevalence of students who smoked increased slightly in 2015 (2.9%) against 2010 (1.8%). It is not easy to compare our findings with those from other countries owing to the different methodologies used. However, the smoking prevalence in our sample was much lower than that reported in a large sample of high school students in Porto, Portugal, which found that 21.5% of the participants were current smokers.^13^ In Ethiopia, a higher prevalence (28.6%) of smoking was found among adolescents.^14^ Similarly, in Chile, the prevalence of smoking was 15.4% among adolescent students aged 13–15 years.^15^ Smoking prevalence among young people in Serbia is the highest in Europe. In fact, 54.7% of teenagers aged 13–15 have already smoked a cigarette, with 31.3% of them having done so by the age of 10.^16^ Our study showed that the majority of the students considered smoking to be harmless to their health. Surprisingly, similar to recent studies conducted in Italy, England and Germany among students,^17,18^ the knowledge of the health risks associated with cigarette smoking was considerably low.

On the other hand, our results showed that in the 2010 study, parents, primary school and TV and radio were the primary sources of information to the students about smoking and its harmful effects compared to the study in 2015. This could be explained either by the differences in the two samples or the place of tobacco control in the primary and middle school curriculum, which may be less marked.

Although it is widely recognised that children smoke their first cigarette while attending primary school, smoking is most likely to begin during adolescence,^19^ when various factors, such as peer pressure, family influence, social class and other psychosocial determinants influence an individual to start and maintain the habit.^20^ Indeed, in the 2015 study, 36.9% of students were exposed to smoking in their home. This prevalence is higher than the GYTS 2010 study, which reports a prevalence of 19.7%. The results of the study argue that the students are influenced by their best friends (32.9%), followed by their parents (27.5%) and finally by their brothers (15%). The inverse case was found in the GYTS 2010 study, which classifies the parents in first place (31%), followed by brothers (22%) and finally friends (12%). Then again, many studies have found that tobacco use is less likely among students belonging to nuclear families, arguing that close contact among parents and children in nuclear families might play a protective role against taking up a risky behaviour such as tobacco use.^21^

## Conclusion

This study reports the positive evolution in the knowledge of students about smoking and its effects. The prevalence of students who smoked increased slightly in 2015. Efforts must be made to reach the long-term objective of reducing the prevalence of smoking among the students. The results of this study suggest the need to address family influences on adolescent smoking and to investigate the introduction of a programme for education and training of students on tobacco dependence prevention, because schools are expected to be the favoured vehicle for health promotion.
